# A portable smartphone-based electrochemical sensing platform for rapid and sensitive detection of creatinine in blood serum†

**DOI:** 10.1039/d5ra03128a

**Published:** 2025-07-15

**Authors:** Rifat Rayhan, Md. Inzamamul Haque Shishir, Md. Abdul Khaleque, Md. Ruhul Amin, Md. Romzan Ali, Mohamed Aly Saad Aly, Shakib Mahmud Ayon, Rahman Saidur, Tan Han Kim, Md. Abu Zaed, Md. Zaved Hossain Khan

**Affiliations:** a Laboratory of Nano-bio and Advanced Materials Engineering (NAME), Jashore University of Science and Technology Jashore 7408 Bangladesh zaved.khan@just.edu.bd; b Department of Chemical Engineering, Jashore University of Science and Technology Jashore 7408 Bangladesh; c Department of Biomedical Engineering, Jashore University of Science and Technology Jashore 7408 Bangladesh; d School of Electrical and Computer Engineering, Georgia Institute of Technology Atlanta GA 30332 USA mohamed.alysaadaly@ece.gatech.edu; e Department of Electrical and Computer Engineering at Georgia Tech Shenzhen Institute (GTSI) Shenzhen Guangdong 518055 China; f Research Centre for Nanomaterials and Energy Technology (RCNMET), School of Engineering and Technology, Sunway University Bandar Sunway 47500 Selangor Darul Ehsan Malaysia; g School of Engineering, Lancaster University Lancaster LA1 4YW UK

## Abstract

Muscle metabolism produces creatinine, a waste product whose levels in the blood and urine are crucial markers of kidney health. Herein, a smartphone-based electrochemical detection strategy was developed to quantify creatinine in human blood serum. Since creatinine was electrochemically inactive, a standard copper solution was added as an electro-activator to produce an electrochemically active creatinine–copper complex. At a pH of 7.4, the creatinine–copper composite was oxidized in a phosphate buffer solution (PBS). Electrochemical oxidation of the free Cu^+^ ion in PBS is tested by the surface modification of Ti_2_C_2_T_*x*_@poly(l-Arg) nanocomposite. The analytical performance of the developed electrochemical sensor was evaluated by differential pulse voltammetry. The developed electrochemical sensor was evaluated using a combination of techniques: electrochemical methods like cyclic voltammetry and electrochemical impedance spectroscopy, morphological analysis with scanning electron microscopy, and structural analysis with attenuated total reflectance Fourier transform infrared spectroscopy and X-ray diffraction. Notably, the developed sensor demonstrated an impressively low detection limit of 0.05 μM and a linear range of 1–200 μM. Moreover, the sensor remarkably exhibited a stable creatinine detection response with an acceptable reproducibility for two two-week periods and demonstrated a robust immunity against interfering molecules. This is the first report on the synthesis of Ti_2_C_2_T_*x*_@poly(l-Arg) nanocomposites and their application in the electrochemical detection of creatinine. This smartphone-based creatinine sensor offers a promising, rapid, and reliable technique for creatinine detection, with potential applications in clinical diagnostics and biomedical research, due to its high sensitivity, selectivity, and portability.

## Abstract 2

Muscle metabolism produces creatinine, a waste product whose levels in the blood and urine are crucial markers of kidney health.

## Graphical abstract

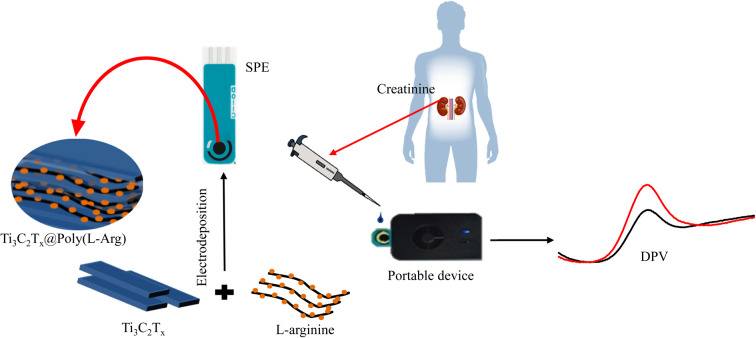

## 1 Introduction

Creatinine (2-amino-1-methyl-5*H*-imidazole-4-one) is a byproduct formed because of the breakdown of creatine phosphate in muscles, and it's filtered by the kidneys.^1^ It is a crucial marker of kidney function: excessive levels of creatinine in the blood or urine can indicate conditions such as glomerulonephritis, diabetic nephropathy, eclampsia, and chronic kidney disease (CKD), which is a global health concern that can lead to renal failure;^2^ low levels of creatinine can be indicative of fluid loss, muscular dystrophy, or liver problems. Therefore, effective measuring and reliable monitoring of creatinine levels is essential in clinical diagnostics. The literature revealed that acceptable blood creatinine concentration levels are within the range of 0.06–0.11 M in men and 0.045–0.09 M in women,^3^ while a concentration up to 0.14 M may be an sign of a syndrome.^4^ Additionally, it was reported that the typical ranges for creatinine concentrations in urine were 0.033–0.225 M for men and 0.0036 to 0.027 M for women,^5^ while ranges less than 0.0018 M suggest hyperthyroidism, anemia and kidney failure.^6^ Additionally, satisfactory creatinine levels in saliva were reported to be in the range of 0.088–0.0265 M, while levels ranged between 0.0167 and 0.4 M recommending renal failure.^7^ Therefore, real-time detection and effective measurement of creatinine levels through portable platforms are essential for clinical diagnostics and home-based care and can play a vital role in human health monitoring.

Portable smartphone-based sensors are small, integrated systems that enable on-site, real-time detection and analysis of certain analytes or environmental factors by fusing a smartphone with a sensing device.^8^ In case of electrochemical sensors, these devices interpret and depict electrochemical data, such as variations in current, voltage, or impedance, that are produced by redox reactions at the sensor interface by using the smartphone's processing power, display, and connection.^9^ In case of electrochemical sensors, these devices interpret and depict electrochemical data, such as variations in current, voltage, or impedance, that are produced by redox reactions at the sensor interface by using the smartphone's processing power, display, and connection.^10^ Integrating smartphone with electrochemical sensor makes it a multipurpose detector with the capability of detecting different molecules. Therefore, many portable electrochemical sensors were developed to detect different substances such as glucose^11^ viruses,^12^ bacteria,^13^ environmental monitoring,^14^ food safety,^15^ and healthcare,^16^ with high sensitivity, selectivity, and instantaneous findings without requiring lab equipment.^17^ Even though creatinine is a crucial biomarker for evaluating kidney function, muscle metabolism, and general health, there are several reported methods used for the detection of creatinine; however, the Jaffe reaction technique is the most common in clinical settings.^18^ Furthermore, interference and sensitivity problems plague spectrophotometric–colorimetric techniques,^19^ and although enzymatic techniques are selective, their cost is high.^20^ Due to their high sensitivity, cost-effectiveness, and suitability for point-of-care diagnostics, electrochemical sensors have recently attracted much attention. These sensors integrate the high sensitivity of electrochemical transducers with the specificity that biological recognition components such as enzymes and antibodies offer.^21^ Electrochemical sensing with both enzymatic and non-enzymatic techniques is prominent in creatinine detection. Although enzymatic-based electrochemical sensors are noted for their selectivity, they face challenges such as stability and reproducibility caused by denaturalization.^22^ Non-enzymatic sensors employing metallic nanomaterials such as copper, silver, and iron offer promising alternatives. These materials form complexes with creatinine, thus enhancing the detection sensitivity.^23^ Such a detection strategy of creatinine enables a wide range of electrochemical-based diagnostic applications.

MXenes (Ti_3_C_2_T_*x*_), two-dimensional materials derived from transition metal carbides, nitrides, and carbonitrides, are gaining significant attention due to their unique properties like high surface area, metallic conductivity, hydrophilicity, and environmental friendliness, making them promising for various applications,^24^ such as electrochemical energy storage,^25–27^ electrocatalysis,^28^ sensors,^29^ and biosensors. Due to its ease of preparation, structural stability, and numerous active sites, it is suitable for various applications,^30^ particularly effective in electrochemical detection, biosensing platforms.^31,32^ Herein, Ti_3_C_2_T_*x*_ composites were mixed with polymers to enhance the electrodes' electrocatalytic activity.^33^ The poly(l-Arg) guanidyl group was involved in hydrogen bonding, providing special properties that interacted constructively with the partial negative charge of Ti_3_C_2_T_*x*_ electrostatically.^34^ Furthermore, the –OH groups of Ti_3_C_2_T_*x*_ can readily interact with the free amine group of P-Arg. Owing to the tunable basal plane oxygen functionalities of Ti_3_C_2_T_*x*_-based materials, they serve as an efficient sensing framework for the targeted identification of bioentities.^35^ Additionally, to improve the thermal, electrical, and optical characteristics of sensing platforms, metallic nanoparticles functionalized with poly(l-Arg) were previously incorporated into various modified electrodes.^36^ Due to their simplicity in preparation, straightforward surface functionalization, and excellent analytical sensitivity, Ti_3_C_2_T_*x*_ decorated poly(l-Arg) structures are among the most investigated and frequently employed in electrochemical sensing systems,^37^ such as an advanced gas sensor with an ultrahigh signal-to-noise ratio. Also, the molecular architecture of Ti_3_C_2_T_*x*_@poly(l-Arg) is inherently connected to its synthesis because of the synergistic interactions between the functional groups (–NH_2_, –COOH) of poly(l-Arg) and the surface terminations (–O, –F, –OH) of Ti_3_C_2_T_*x*_.^38^ These molecular interactions can be optimized to improve the composite's electrical conductivity, structural integrity, and sensing capabilities by carefully adjusting synthesis parameters such as pH, temperature, and reaction time.^39^ As a result, the preparation technique guarantees the material's functional performance, while the molecular design controls the assembly process.

In this study, a novel detection system for electrochemical sensors intended for creatine monitoring was designed and developed, employing a smartphone-based system *via* a co-reaction technique. The major components of the developed sensing platform were a smartphone, a handheld detector, screen-printing electrodes (SPE), and creatine molecules with a substance-sensitive component. A smartphone application controls the system, processes data, and displays the outputs in real-time. To maximize sample optimization and detection while concurrently sending the gathered data to the smartphone, a handheld detector was created. As a result, a new nanocomposite electrochemical sensor, Ti_3_C_2_T_*x*_@poly(l-Arg), was effectively used for the first time to establish a smartphone-based technique for detecting creatinine in real blood serum. With a detection limit of 0.05 μM and a range of 1 to 200 μM, a strong linear relationship was obtained. The optimal pH for the sensor is 7.4. Moreover, it is not affected by the presence of Na^+^, K^+^, Cl^−^, PO_4_^3−^, ascorbic acid, uric acid, urea, and glucose. Additionally, the level of creatinine in human blood serum with a low detection limit was ascertained by reviewing and comparing the current work to the previously reported creatine sensing platforms. This is the first report on the synthesis of Ti_2_C_2_T_*x*_@poly (l-Arg) nanocomposites and their application for the electrochemical determination of creatinine in human blood serum.

## 2 Materials and methods

### 2.1. Chemicals and instruments

In this investigation, every reagent utilized was analytically pure and used instantaneously, without additional purification. PBS was acquired from Sigma-Aldrich in China, and l-Arg was purchased from Aladdin's Reagents in Shanghai, China. A 100 mM PBS solution with a pH of 7.4 served as the electrolyte solution for all electrochemical experiments. Ti_3_AlC_2_ powder (MAX phase) was purchased from the Nano-Materials and Energy Technology at Sunway University, Malaysia. l-Arg (≥98% purity, molecular weight 174.20 g mol^−1^, CAS No. 74-79-3) was purchased from Sigma-Aldrich and used without further purification. Creatinine was bought from Sisco Research 130 Laboratories Pvt. Ltd. The Fluka standard copper solution for atomic absorption spectrometry (AAS), standardized by Sigma-Aldrich, was purchased. All preparations used ultrapure water (Evoqua Type-I, Germany) with a resistivity of less than 18.2 MΩ. Electrochemical investigations were performed on the electrochemical workstation Sensit Smart (Palm Sens B.V., Netherlands) equipped with Redmi Note 12 and a 3 mm-diameter SPE. A scanning electron microscope (SEM) of the ZEISS GeminiSEM500 model was used to examine the surface morphology of the functionalized electrodes. The crystal structure investigations were assessed using X-ray diffraction (XRD) patterns (RINT2200, Rigaku, Japan) and attenuated total reflectance-Fourier transform infrared (ATR-FTIR, NICOLET iS20) spectroscopy in the 2*θ* range of 20–80° at a scan rate of 0.02° S^−1^ and room temperature.

### 2.2. MXene synthesis

First, 15 mL of hydrochloric acid at a concentration of 9 M and 1.6 g of lithium fluoride were used to etch the powder form of the MAX phase (Ti_3_AlC_2_) in 5 mL of deionized water. This mixture contains high-density polyethylene and is stirred magnetically for 24 hours. The resulting solution underwent a washing process through centrifugation at 4200 rpm for 7 minutes, followed by decantation and water dispersion. Repeated washing cycles neutralized the acidic solution and eliminated contaminants. The final solution (pH 6.0) was centrifuged (10 000 rpm, 45 minutes) to yield a concentrated MXene paste, which was subsequently dried in a vacuum oven (60 °C, 12 hours), as previously reported.^40^

### 2.3. Fabrication of the sensor electrode

**Scheme 1:**
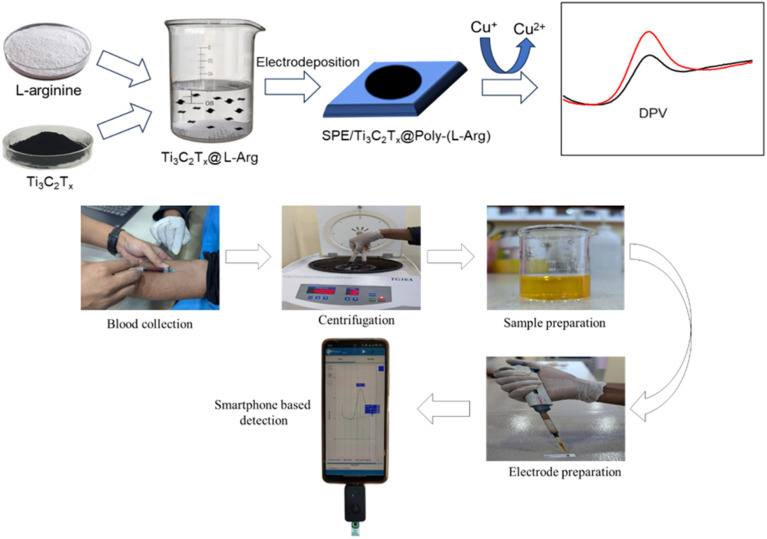
Schematic representation of the fabrication of SPE/Ti_3_C_2_T_*x*_@poly(l-Arg) and the smartphone-based electrochemical detection of creatinine *via* Cu^2+^ complexation and interaction with the modified electrode surface.

Using 0.05, 0.3, and 1.0 μm alumina powder on a micro cloth polishing pad, the SPE working electrode (3 mm diameter) was meticulously polished. After being cleaned with ultrapure water, the polished electrode was sonicated for three minutes using deionized water, diluted nitric acid, and absolute ethanol. Inert nitrogen gas was then used to dry the resultant working surface. The electrodeposition technique was then used to modify the working electrode with Ti_3_C_2_T_*x*_@poly-(l-Arg) composite. The composite was deposited on the surface of SPE for 12 cycles at a scan rate of 50 mV s^−1^ using the CV method, with an electro-deposition potential range of −2.2 to 2.2 V *vs.* Ag/AgCl. After a thorough rinsing of the deposited electrode, the modified electrode was given the designation of SPE/Ti_3_C_2_T_*x*_@poly(l-Arg), as shown in the upper half of Scheme 1.

### 2.4. Electrochemical detection of creatinine

In this work, an approach was employed to investigate the electrochemical detection of creatinine, serving as a vital biomarker indicating renal function. To balance precision and computational efficiency, a 10 ppm standard Cu solution was chosen for the reaction in this investigation. This concentration was chosen to ensure accurate measurement and simplified analysis, which made it perfect for experimental consistency and real-world application, even though copper concentration is proportionate to the reaction result, as shown in Fig. S1† (ESI†), following the previously reported copper–creatine complex reaction mechanism.^41^ Through hydrogen bonding, the guanidinium side chains of poly(l-Arg) may establish substantial relations with the carbonyl moiety of creatinine, whereas the amine group has affinities for the –OH and –O functional groups on the surface of Ti_3_C_2_T_*x*_. The electrode's selectivity and sensitivity for creatinine are greatly increased by this dual-modal interaction architecture. The molecular architecture was carefully constructed to take advantage of the special functional groups that make creatinine unique, rather than being random, to assure efficient collection and signal transduction inside the electrochemical sensor. This method effectively combines material design with biosensing capabilities to address the unique molecular properties of the target analyte.

Here, voltametric methods, namely differential pulse voltammetry (DPV), were used to perform electrochemical experiments. To provide the best possible detection of the copper–creatinine structure, the voltammetry measurements were taken within a potential window ranging from −300 to 600 mV. The ability of the proposed sensor to accurately identify and quantify creatinine in the presence of potential interferences such as urea, uric acid, glucose, ascorbic acid, and various salts was systematically investigated. These interferent-containing solutions were introduced into the PBS matrix, and their impact on the creatinine detection system's electrochemical response was rigorously evaluated. The sensor's selectivity was assessed through chronoamperometric measurements conducted at a fixed potential of 2.5 V over 500 seconds. At *t* = 190 s, creatinine was introduced into the electrolyte solution, which generated a well-defined current response of 18 μA. This distinct signal emergence demonstrates the sensor's specific recognition capability for creatinine amidst the background electrolyte. Through experimental design and rigorous evaluation, valuable insights into the development of robust electrochemical sensing platforms for clinical diagnostics are provided in this study. A schematic diagram displaying the detection flow for creatinine is shown in the lower half of Scheme 1.

## 3 Results and discussion

### 3.1. Surface morphology and functional group analyses

**Fig. 1:**
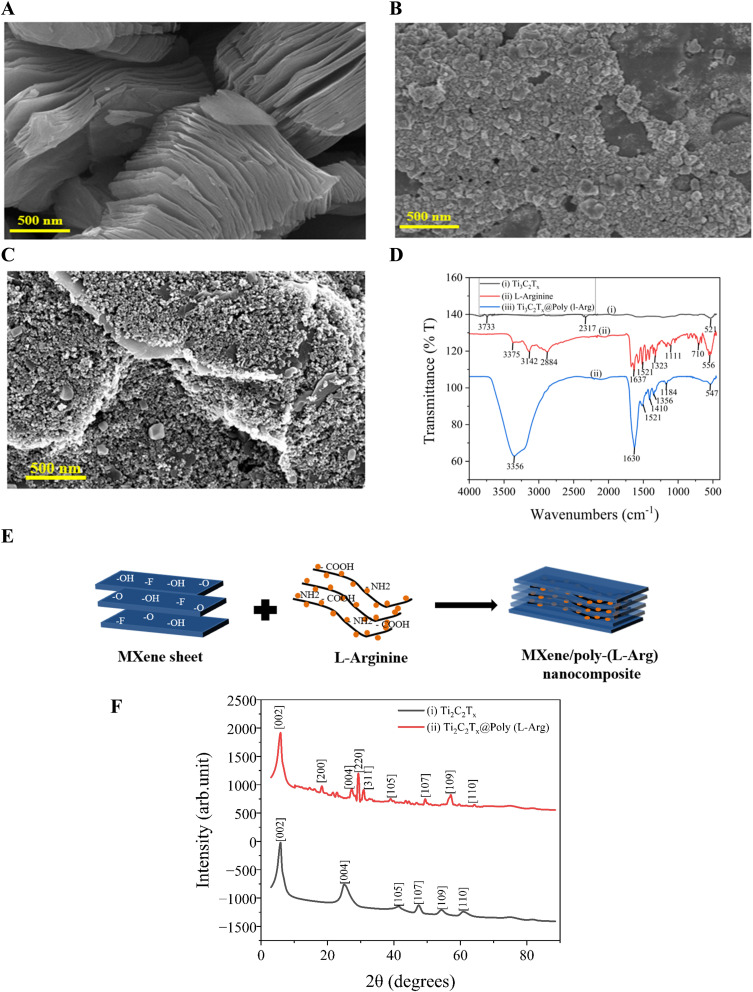
SEM image of (A) Ti_3_C_2_T_*x*_; (B) l-Arg; (C) Ti_3_C_2_T_*x*_@poly (l-Arg); (D) ATR-FTIR spectrum of Ti_3_C_2_T_*x*_, l-Arg, and Ti_3_C_2_T_*x*_@poly (l-Arg); (E) the electropolymerization process creates an interconnected nanofibrillar network of poly-(l-Arg) on MXene sheets, contributing to the observed surface roughening; (F) XRD spectrum of Ti_3_AlC_2_; and Ti_3_C_2_.

The surface morphology, chemical structure, functional groups, and elemental composition study of MXene (Ti_3_C_2_T_*x*_) preparation are presented in the SEM images, FTIR spectrum, and XRD study shown in Fig. 1(A–F). The distinctive layered and smooth sheet-like structure of Ti_3_C_2_T_*x*_, consistent with its exfoliated 2D shape, is shown in Fig. 1(A). Fig. 1(B) presents the granular particulate morphology of pure l-Arg. Fig. 1(C) shows an altered and roughened surface structure, indicating that poly-(l-Arg) was successfully polymerized and bound to the MXene sheets. The observed morphological change from smooth (MXene) to a textured (composite), further demonstrates how well the polymeric l-Arg coating functionalizes the Ti_3_C_2_T_*x*_ surface. The electropolymerization process creates an interconnected nanofibrillar network of poly-(l-Arg) on MXene sheets. This structural transformation results from specific interactions between the polymer's functional groups (–NH_2_, –COOH) and MXene's surface terminations (–O, –F, –OH), collectively contribute to the observed surface roughening shown in Fig. 1(E)

Fig. 1(D) displays the FTIR spectrum of Ti_3_C_2_T_*x*_ MXene, l-Arg and Ti_3_C_2_T_*x*_@poly-(l-Arg), showing several distinctive absorption bands. For Ti_3_C_2_T_*x*_ the C=O stretching from leftover oxygen terminations is shown by the peak at ∼1630 cm^−1^. Between 500 and 700 cm^−1^, characteristic Ti–O oscillations are noted. The successful removal of Al from the MAX phase during etching is confirmed by the lack of prominent peaks in the 1000–1400 cm^−1^ area.^42^ The stretching vibrations of NH and OH are represented by a wide absorption band in the wavelength range of 3375–2884 cm^−1^. Additionally, the stretching vibrations of CH are attributed to absorption bands between 2850 and 2960 cm^−1^.^43^ The bending vibrations of NH_2_ are linked to the band that spans from 1521 to 1630 cm^−1^. Other prominent peaks include 1410 cm^−1^, reflecting the bending vibration of CH_3_, and 1323 cm^−1^ is associated with the deformation vibrations of CH and NH. Furthermore, the symmetrical bending vibration of CH_3_ is given a peak at 1410 cm^−1^, whereas a peak indicates the deformation vibration of CH at 1356 cm^−1^. The peak represents the bending vibration of OH at 1323 cm^−1^, while the stretching vibration of CO and the deformation vibration of OH was assigned to the peak at 1184 cm^−1^, and 1111 cm^−1^ for CO stretching, 710 cm^−1^ for CNH stretching, and 1130 cm^−1^ for CN stretching are other important peaks. Additionally, following earlier research, the peaks at 710 cm^−1^ and 556 cm^−1^ show the bending vibrations of COO and CO, respectively, while the peaks at 545 cm^−1^ show the deformation vibration of CH.^44–49^ When l-Arg establishes a covalent bond with Ti_3_C_2_T_*x*_, the characteristic peaks of both MXene and l-Arg often shift. This might be due to the creation of bonds such as Ti–O or Ti–N or even direct interaction with terminal functional groups such as –OH, –F, or –O on Ti_3_C_2_T_*x*_. It is evident from the Ti_3_C_2_T_*x*_ and l-Arg spectra that the spectrum of l-Arg, which is covalently linked to Ti_3_C_2_T_*x*_, was preserved.

Additionally, Fig. 1(F) presents the XRD patterns of Ti_3_C_2_T_*x*_ and Ti_3_C_2_T_*x*_@poly-(l-Arg). In a previous study, the crystallinity and structural order of Ti_3_C_2_T_*x*_ were altered due to the removal of Al layers at 39.2° *via* HF etching, resulting in the formation of Ti_3_C_2_T_*x*_.^50^ The XRD pattern of Ti_3_C_2_T_*x*_ exhibits characteristic peaks corresponding to the [004], [105], [107], [109], and [110] planes at 24.76°, 41.28°, 47.32°, 54.28°, and 60.86°, respectively. In contrast, the Ti_3_C_2_T_*x*_@poly(l-Arg) composite displays additional diffraction peaks at 18.3°, 29.3°, and 30.96°, which can be attributed to the [200], [220], and [311] planes, respectively. These findings, in conjunction with FTIR analysis, confirm the successful formation of the Ti_3_C_2_T_*x*_@poly-(l-Arg) composite as shown in Fig. 1(D).

**Fig. 2:**
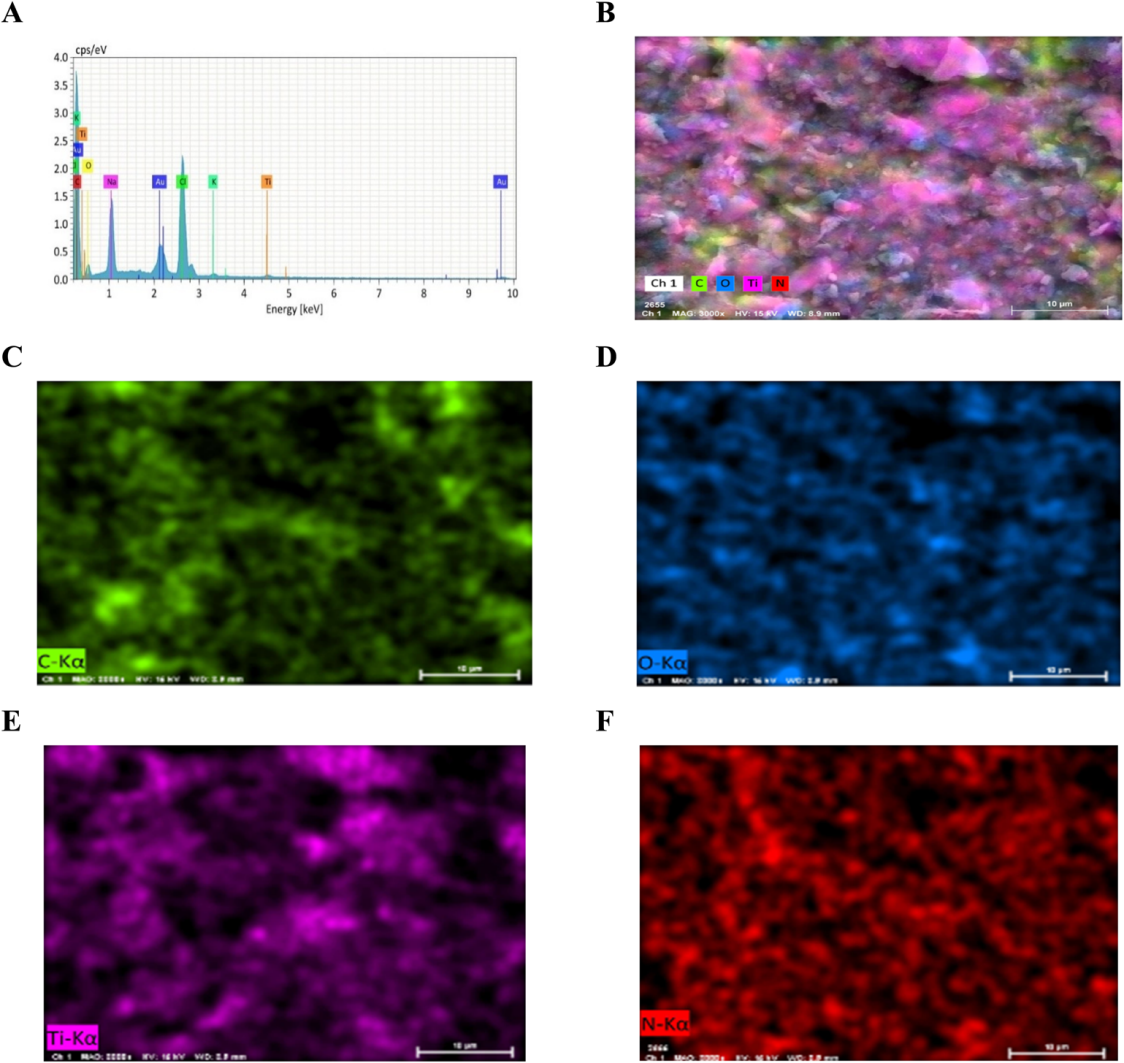
EDX spectrum (A) showing 68.98% C, 7.70% N, 4.50% O, 0.62% Ti and 18.22% are Na, Cl, K, Au; EDX elemental mapping of (B) C, O, Ti, N; (C) C; (D) O; (E) Ti, (F) N.

The elemental composition of the changed electrode surface was shown by the EDX spectrum Fig. 2(A), which confirmed the successful synthesis of the Ti_3_C_2_T_*x*_@poly(l-Arg) composite. The intended components were carbon (C, 68.98%), nitrogen (N, 7.70%), oxygen (O, 4.50%), and titanium (Ti, 0.62%). Notably, the Ti_3_C_2_T_*x*_ MXene structure is responsible for titanium and oxygen, whilst the l-Arg component is responsible for the high carbon and nitrogen content. Furthermore, trace levels of potassium (K), sodium (Na), and chlorine (Cl) were found in total (18.22%), most likely as a result of electrolyte interactions or leftover contaminants during production. The conductive coating used to prepare the EDX sample is what causes the presence of gold (Au). Additionally, the collective and individual elemental mapping with color visualization shown in Fig. 2(B) and (C–F), verified the coherence and well integration of the composite.

### 3.2. Electrochemical polymerization

As seen in Fig. S2,† the modified Ti_3_C_2_T_*x*_@poly(l-Arg) electrode underwent cyclic voltammetry (CV) optimization using twelve consecutive scans (−2.0 to +2.0 V, 100 mV s^−1^). The voltammograms showed steady electrochemical behavior across cycles, with incremental increases in the reduction currents at 0.0 V (confirming efficient composite immobilization) and oxidation currents at +2.0 V (showing continuing polymer development). The best surface coverage and charge transfer properties were determined by this methodical tuning, guaranteeing dependable sensor performance for creatinine detection. The repeatable CV profiles demonstrate the electrode's potential for accurate biomedical diagnostics and healthcare monitoring applications while validating its strong sensing capabilities.

### 3.3. Electrochemical characterization

**Fig. 3:**
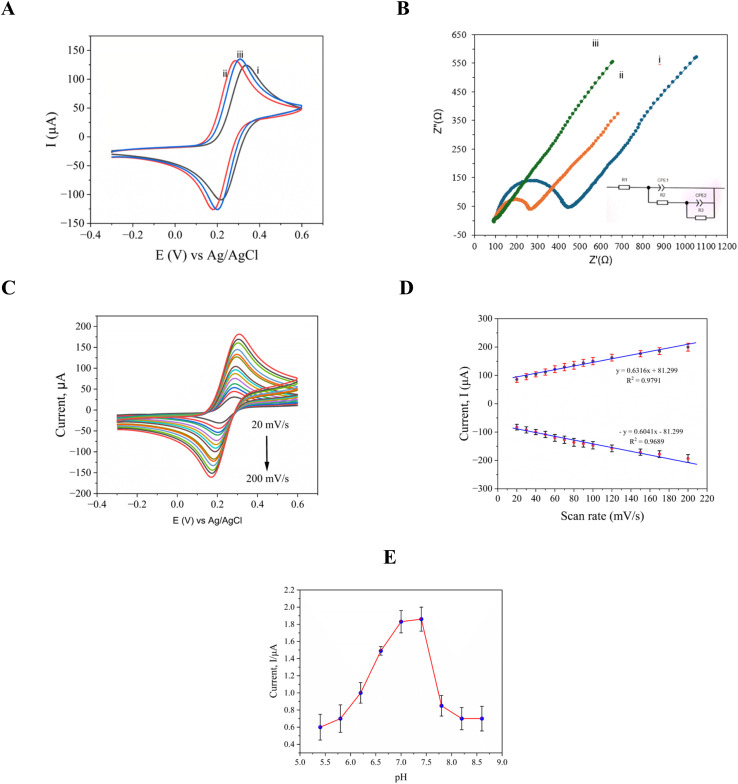
Cyclic voltammograms (A); and the Nyquist plot (B); of (i) bare SPE, (ii) SPE/poly(l-Arg), (iii) Ti_3_C_2_T_*x*_@poly(l-Arg)/SPE in 0.1 M KCl including 5.0 × 10^−3^ M [Fe(CN)_6_]^3−/4–^; and (C) Ti_3_C_2_T_*x*_@poly(l-Arg)/SPE test at scan rate of 20 to 200 mV s^−1^ in 0.1 M KCl including 5.0 × 10^−3^ M [Fe(CN)_6_]^3−/4–^; (D) anodic and cathodic linearity of current at scan rate; and (E) DPV response of 1.0 × 10^−4^ M of CRE at a pH of 5.4, 5.8, 6.2, 6.4, 7, 7.4, 7.8, 8.2, and 8.6.

Cyclic voltammetry (CV) was used to assess the electrochemical performance of the developed sensor in a redox probe solution that included 100 mM KCl and 5.0 mM [Fe(CN)6]^3−/4−^. The behavior of the sensor at various modification phases, such as post-immobilization and hybridization, is depicted in Fig. 3(A). With a peak-to-peak potential separation (Δ*E*_p_) of 120 mV, and oxidation and reduction peaks at 0.338 V and 0.218 V, respectively, the pristine electrode demonstrated a well-defined redox response under neutral circumstances as presented in Fig. 3(A)(i), demonstrating effective electron transfer kinetics. When compared to the bare SPE, the electrocatalytic activity of the modified electrode was greatly increased by the addition of l-Arg (SPE/poly-(l-Arg)), as seen by the improved redox peak currents and a decreased ΔEp of 114 mV (oxidation at 0.295 V, reduction at 0.181 V) shown in Fig. 3(A)(ii). This enhancement implies that the l-Arg layer promotes quicker electron transfer, most likely as a result of its advantageous interfacial characteristics and conductive polymeric structure. By adding MXene to the l-Arg-modified electrode, more improvement was made as illustrated in the results presented in Fig. 3(A)(iii). The resultant composite highlighted MXene's remarkable electrochemical qualities with a smaller Δ*E*_p_ (108 mV, with peaks at 0.308 V and 0.200 V) and a sharper oxidation peak. This increase is due to the synergistic enhancement of charge transfer kinetics by MXene's high electrical conductivity, large surface area, many surface functional groups, variable hydrophilicity, and effective ion intercalation capabilities.

To evaluate the performance of the sensor, detailed electrochemical impedance spectroscopy (EIS) studies were conducted using a 5 μM [Fe(CN)_6_]^3−/4−^ solution with 0.1 M KCl as the redox probe. The Nyquist plots in Fig. 3(B) reveal significant differences in electron transfer efficiency between the three electrode configurations. The results of the unmodified electrode, shown in curve (i) of Fig. 3(B) exhibited substantial charge transfer resistance (*R*_ct_ = 372 Ω^−1^), evident from its large semicircular profile, indicating restricted electron flow at the electrode–electrolyte interface. Modification with poly(l-Arg) reduced this resistance by 51% (*R*_ct_ = 181 Ω^−1^), as shown in the results presented in curve (ii), demonstrating improved charge transfer capabilities through the enhanced surface conductivity and favorable electrostatic interactions with the redox probe. The most dramatic improvement was achieved by the Ti_3_C_2_T_*x*_@poly(l-Arg) composite modification, as shown in the results presented in curve (iii), thus resulting in an exceptionally low *R*_ct_ of just 9 Ω^−1^, a 98% reduction compared to the bare electrode. This modification virtually eliminated the characteristic semicircular impedance profile, signaling transition to diffusion-controlled kinetics. The combination of MXene's outstanding electrical conductivity with poly(l-Arg) interfacial modification properties created an optimal environment for rapid electron transfer, significantly boosting the sensor's electrochemical performance.

Additionally, the proposed equivalent circuit accurately represents the electrochemical processes observed in the conducted EIS measurements. The model consists of three main elements: (1) *R*_1_, accounting for the inherent electrolyte resistance; (2) a parallel *R*_2_–CPE_1_ network modeling the primary charge transfer dynamics at the electrode interface; and (3) an additional *R*_3_–CPE_2_ pair capturing secondary interfacial effects. This configuration effectively describes the performance enhancements achieved through electrode modification. While the unmodified electrode shows substantial charge transfer resistance (*R*_2_ = 372 Ω^−1^), the functionalized electrodes demonstrated progressively lower values. Most notably, the Ti_3_C_2_T_*x*_@poly(l-Arg) modified electrodes achieved an exceptionally low *R*_2_ of just 9 Ω^−1^, reflecting the superior conductivity and efficient charge transfer characteristics of the developed modified electrodes.

The reaction kinetics of the modified electrode were examined using cyclic voltammetry at several scan speeds with a range of 20–200 mV s^−1^. The anodic and cathodic peak currents rose as the scan rate increased. Around 0.19 V and 0.3 V, the oxidation and reduction peaks were visible. Fig. 3(C) displays the cyclic voltammogram at various scan rates. Fig. 3(D) demonstrates how the currents at the cathodic (reduction) and anodic (oxidation) peaks change with variations in the scan rate. In the context of creatinine sensing, the graph highlights how the scan rate affects the currents at these two peaks, which are essential for measuring creatinine levels. The target molecules' linear connection between Ip and scan rate is also displayed in Fig. 3(D). *I*_pa_ (μA) = 0.6316*x* + 81.299 is the regression equation for creatinine oxidation current, with a coefficient of regression of *R*^2^ = 0.9791. The regression equation for reduction current is −*I*_pc_ = 0.6041*x* − 81.299, with a coefficient of *R*^2^ = 0.9689.

The peak potential and current were both impacted by the pH of the assisting electrolyte, which was a critical factor in the creation of electro-oxidation at the modified electrode. The ideal pH value between 4.0 and 8.0 in a 0.01 M PBS solution for 100 μM creatinine was examined using the differential pulse voltammetry (DPV) technique, and the results are presented in Fig. 3(E). The peak current of creatinine grows steadily as the pH level of the oxidation process rises from 4.0 to 7.4. It reaches its maximum current at the pH level of 7.4, and it then falls from 7.4 to 8.0. Creatinine deprotonates beyond pH 7.4, and since there aren't enough protons, electrochemical processes are difficult. Creatinine has a p*K*_a_ value of 12.3, at a pH of 7.4, and it was calculated by the Henderson–Hasselbalch equation. The analytes become protonated as the pH rises from 4.0 to 7.4, resulting in a noticeably increased oxidation peak. Nevertheless, the analytes get deprotonated and reduce the bulk solution's absorption rate on the working electrode as the pH range shifts from 7.4 to 9.0. The analytes might be oxidized in the air within that pH range as a result of the oxidation current decreasing as the pH rises to 7.4. Consequently, a pH of 7.4 was used for the creatinine assessment. These evaluations showcase the modified electrode's potential as a precise and selective platform for creatinine detection, highlighting its suitability for diverse analytical applications and its ability to detect creatinine at 300 with high sensitivity and specificity.

### 3.4. Electrochemical detection of creatinine

**Scheme 2:**
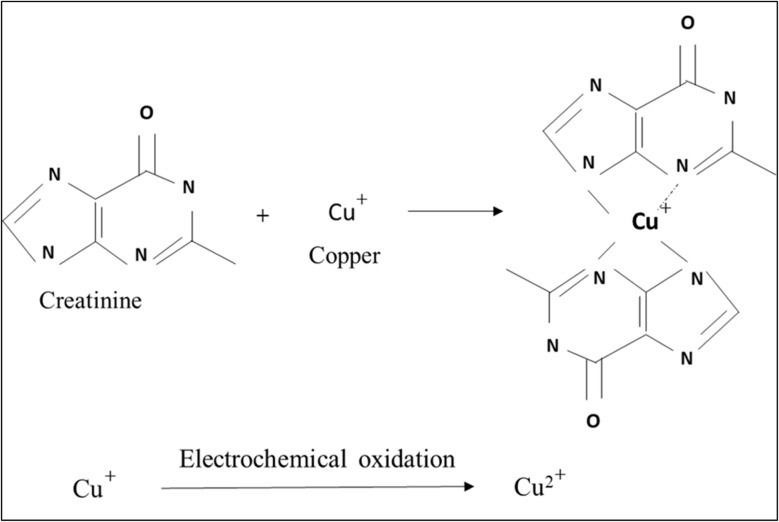
Copper–creatinine complex formation and the electrochemical oxidation of the unreacted Cu^+^ for quantification of creatinine.

In this analytical approach, a standardized solution of copper(i) (Cu^+^) is introduced into a sample containing creatinine. The Cu^+^ ions reacted with creatinine to form a stable, electrochemically inert complex, [Cu(creatinine)_2_(H_2_O)_2_]^2+^.^51^ Any excess (unreacted) Cu^+^ remaining in the solution was subsequently oxidized to Cu^2+^*via* electrochemical means. By measuring this oxidation signal, the concentration of creatinine can be determined indirectly. The method was validated for creatinine concentrations spanning from 1.0 to 200 μM, using a fixed Cu^+^ concentration of 0.01 μM. This approach provides a reliable and sensitive means of detecting creatinine within the specified range. And the reaction mechanism is given below in Scheme 2.

**Fig. 4:**
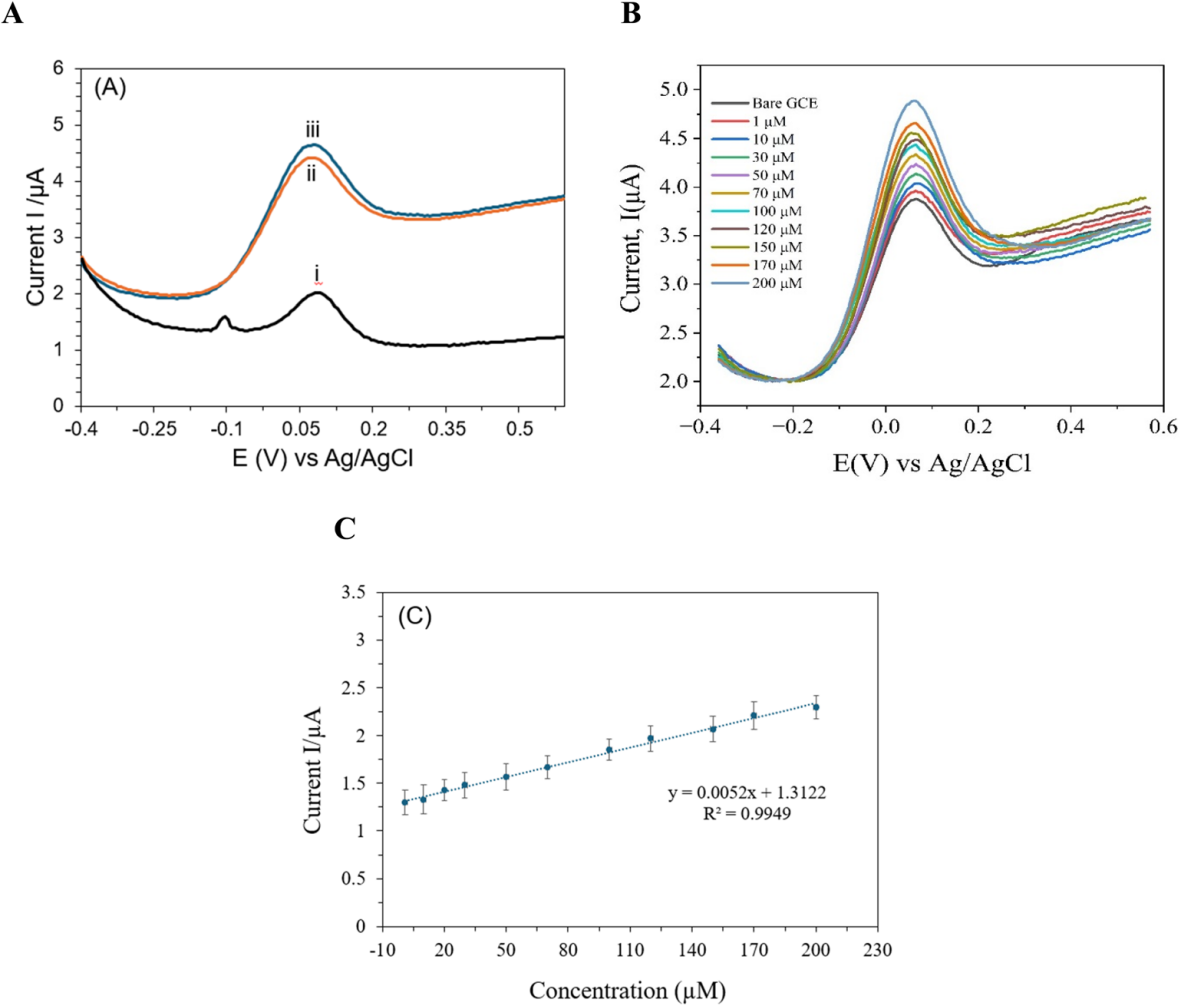
DPV of (A) 100 μM of CRE by (i) bare SPE, (ii) SPE/l-Arg, (iii) SPE/MXene/poly-(l-Arg); (B) 1–200 μM in 0.1 M PBS (pH 7.4) buffer solution at the SPE/MXene/poly-(l-Arg); (C) the corresponding calibration plot of Fig. 3(B).

DPV was employed to extensively examine the modified electrode's electrochemical activity in the presence of creatinine over a broad potential range. A neutral pH level of 7.4 and room temperature were maintained throughout the exact execution of all experimental procedures in a 0.01 M PBS solution. To ensure both precision and repeatability, the DPV studies were carried out using precise parameters: a pulse duration of 300 ms, a pulse width of 0.025 ms, and a pulse amplitude of 50 mV. A peak current at a 0.07 V oxidation peak potential was observed, as shown in Fig. 4(A). The calibration curve, in Fig. 4(C), of creatinine shows linearity from 1–200 μM (*R*^2^ = 0.9914). Fig. 4(B) presents clear anodic peaks corresponding to different concentrations of creatinine, reflecting the sensitivity of the proposed electrode. The limit of detection (LOD) achieved by the present electrode stands at 0.05 μM, underscoring a remarkable sensitivity for the detection of creatinine. In the designated range of 1–200 μM for the detection of creatinine in blood serum, the device showed a linear response, displaying a dependable and consistent performance across this concentration range. Although an increase in current was noted with greater creatinine concentrations outside of this range, the reaction has not yet been completely modeled or described.

A comparative analysis evaluating the findings of the current work with respect to the previously reported studies on electrochemical detection of creatinine is presented and summarized in Table 1. Ti_3_C_2_T_*x*_@poly(l-Arg) has a LOD of 0.050 μM, which is far lower than the majority of the reported materials, as shown in Table 1. Ti_3_C_2_T_*x*_@poly(l-Arg) outperforms SPE/Cu NPs (LOD = 0.0746 μM) and Pt-MEA (LOD = 0.059 μM), as seen in Table 1. This suggests that the developed composite introduced in this work can detect creatinine at extremely low quantities with greater sensitivity than previously reported composites. Similarly, the synthesized composite has a linear range of 1 to 200 μM, making it appropriate for the detection of creatinine in both pathological and normal physiological conditions (50 to 120 μM). However, certain materials have higher LOD and larger linear ranges, such as SPE/Fe^3+^ p-a (100–6500 μM) and enzyme@CS/PB/MXene@AuNP/SPCE (30–4000 μM). It reflects how well the developed composite balances between dynamic range and sensitivity (with the ability to capture both faint and strong signals without distortion or loss of detail). Additionally, the combination of Ti_3_C_2_T_*x*_ (MXene) and poly(l-Arg) is considerably novel and effectively takes advantage of both materials, since MXene offers superior surface area and conductivity, and poly(l-Arg) improves selectivity and increases stability and biocompatibility.

**Table 1:** Comparison of various electrode performances for creatinine detection^a^

Electrode material	Electrochemical technique	Linear range (μM)	LOD (μM)	Ref.
SPE/Fe^3+^ p-a	DPV	100–6500	43.000	52
GCE/TMSPMA-GO-*co*-HEMA/MMA	DPV	44.2–265.21	16.600	53
SPE/Cu NPs	CV	1–180	0.39	54
Pt-MEA	LSV	0.0–5.00	0.059	55
Enzyme@CS/PB/MXene@AuNP/SPCE	DPV	30–4000	10.000	56
g-SPE/CuNPs	DPV	5–125	2.300	57
β-PbO_2_/CNT	OCP	10–400	0.060	58
SPE/Ti_3_C_2_T_*x*_@poly(l-Arg)	DPV	1–200	0.050	This work

a Abbreviations: Fe^3+^ p-a: iron(iii) absorbs within the paper; TMSPMA-GO-*co*-HEMA/MMA: trimethyl silane propyl methacrylate-GO copolymerized with 2-hydroxy methacrylate/methyl methacrylate; MEA: microelectrode arrays.

### 3.5. Integration of smartphone-based system for creatinine detection

This work introduces a smartphone-based portable platform for point-of-care creatinine detection, offering a cost-effective and user-friendly alternative to traditional spectrophotometric or benchtop electrochemical systems. By connecting a USB-enabled potentiostat to a custom-designed smartphone application, real-time voltammetric analysis with rapid detection (<1 minute) and minimal sample volume requirements (<50 μL) was enabled, making it particularly suitable for field testing and remote healthcare applications. Unlike enzyme-dependent assays that necessitate cold storage to prevent protein degradation, the present enzyme-free approach ensures long-term stability at ambient temperatures, eliminating logistical challenges in resource-limited settings. A comparison of the current smartphone-based sensor to other previously reported sensing platforms with respect to sensitivity, cost, portability enzyme stability, detection time and interference issues, is summarized in Table 2.

**Table 2:** Comparison of the current smartphone-based sensor to other previously reported sensing platforms

Method	Sensitivity	Cost	Portability	Enzyme stability	Detection time	Interference issues	Ref.
Jaffe reaction	Moderate	Low	No	N/A	∼30 min	High	59
Enzymatic methods	High	High	Limited	Poor	∼10–20 min	Low	60
Lab-based EC sensors	High	Medium	No	Good	∼5–10 min	Medium	61
Smartphone-based sensor	High	Low	Yes	Stable (non-enzymatic)	<1 min	Low	This work

### 3.6. Stability, reproducibility, and selectivity of the proposed sensor

The proposed sensor showed a stable out throughout the testing time. The sensor produced a maximum current output of 1.4 μA on the day of manufacturing. A 24 hours later, the current output of the sensor was 1.31 μA and remained remarkably consistent, suggesting no destructive deterioration. Additionally, the sensor continued to produce a steady 1.09 μA current output even after 14 days. This long-term performance consistency highlights the stability of the proposed sensor, as shown in Fig. S3 (ESI).†

Critical metrics, including selectivity, pH sensitivity, and repeatability, were methodically evaluated to fully assess the improved electrode's performance. A series of repeated differential pulse voltammetry measurements using the same electrode were used to assess reproducibility, a crucial metric for assessing the dependability of the sensor. The DPV measurements showed no variation in the current response across different analyte concentrations. These results indicate an acceptable repeatability, confirming the potential of the proposed sensor for precise and reliable detection of creatinine suitable for analytical and clinical applications, as shown in Fig. S4 (ESI).†

For 100 μM creatinine detection, the reaction of the modified electrode to a variety of possible interfering chemicals and metal ions, here 100 fold of Na^+^, K^+^, Cl^−^, PO_4_^3−^ and 1 fold of urea, glucose, ascorbic acid, and uric acid, offered a full evaluation of the sensor's selectivity. The oxidation signals corresponding to creatinine demonstrated the remarkable selectivity of the sensor, which remained steady and unaffected in the presence of all investigated interferents. The accuracy and dependability of the developed sensor for creatinine detection in challenging conditions were further highlighted by the variation in selectivity, which was less than 0.5%. This result reflects the potential of the developed sensor for precise and interference-free analysis in practical applications, as shown in Fig. S5 (ESI).† The chronoamperometric response in Fig. S6 (ESI)† shows that the developed sensor exhibits remarkable selectivity in the presence of common physiological interferents. In particular, with the applied potential, a very little cross-reactivity was observed with 100 fold of Na^+^, K^+^, Cl^−^, PO_4_^3−^, and 1 fold of urea, glucose, ascorbic acid, and uric acid, confirming strong selectivity against possible interfering species.

### 3.7. Real sample analysis

The efficacy of the proposed sensor was further assessed through the determination of creatinine in human blood serum samples. A 10 mL of serum solution was prepared by combining 100 μL of serum with an equal volume of copper standard solution. Subsequently, spike tests were conducted by adding 0.01, 0.5, 0.1, and 0.12 mM of creatinine to the prepared serum solution. The analysis of real samples yields recovery rates between 100.11% and 102.00%, as summarized in Table 3. These results unequivocally demonstrate the capability of the developed sensor to accurately detect creatinine in human serum samples, emphasizing its high potential of being use in clinical settings.

**Table 3:** Practical determination of creatinine in human serum samples (*n* = 4)

Creatinine concentration (μM)		Recovery (%)	Relative standard deviation (RSD) (%)
Added	Found		
10	10.5	105.00%	3.34%
10	10.3	103.00%	
10	9.7	97.00%	
50	53.42	106.84%	5.94%
50	51.5	103.00%	
50	47.65	95.30%	
100	103.42	103.42%	3.24%
100	98.19	98.19%	
100	104.39	104.39%	
120	117.20	97.67%	2.04%
120	120.00	100.00%	
120	123.20	123.67%	

## 4 Conclusion

A portable smartphone-based electrochemical sensing platform designed for the electrochemical determination of creatinine in blood serum medium is presented in this work. The developed functionalized electrode displayed a linear association between the peak current and the concentration of creatinine, ranging from 1.0 to 200 μM, and further offered a detection limit of 0.05 μM. This indicates the designed electrode's remarkable sensitivity for creatinine detection. Additionally, the proposed sensing platform provides an affordable solution to achieve desirable selectivity, reproducibility, and sensitivity to the change in pH level, thus enhancing the overall efficiency. Remarkably, the developed sensing platform yields great recovery rates and demonstrates outstanding dependability in detecting creatinine in real samples, confirming great potential for use in clinical settings. Furthermore, this is the first to report on the synthesis of Ti_2_C_2_T_*x*_@poly(l-Arg) nanocomposites and their application as a sensing platform for electrochemical quantification of creatinine in human blood serum.

## Ethics approval

Healthy blood samples were collected from healthy donors, who provided written informed consent for this study according to the Declaration of Medical Services. The blood drawing and experimental procedures were performed in accordance with the Guidelines for Care and Use of Human Samples of Jashore University of Science and Technology (JUST) and approved by the Medical Ethics Committee of Jashore-7408, Bangladesh.

## Author contributions

Rifat Rayhan: electrode modification, data collection, and results analysis; Md. Inzamamul Haque Shishir: data collection, real sample preparation, and analysis; Md. Abdul Khaleque: characterization; Md. Ruhul Amin: data collection, set-up smartphone experiment, and results discussion; Md. Romzan Ali: electrode modification; Mohamed Aly Saad Aly: conceptualization, methodology, data analysis and discussion, supervision, project management, reviewing and editing; Sakib Mahmud Ayon: writing manuscript first draft; Rahman Saidur: MXene synthesis and characterization; Kim Han Tan: results analysis and discussion; Md. Abu Zaed: reviewing and editing; Md. Zaved Hossain Khan: conceptualization, supervision, project management.

## Conflicts of interest

The authors declare that there is no conflict of interest.

## Supplementary Material

RA-015-D5RA03128A-s001

## Data Availability

The datasets used and analyzed during the current study are available from the corresponding author upon reasonable request.
