# Supplementary material for: A portable smartphone-based electrochemical sensing platform for rapid and sensitive detection of creatinine in blood serum
Source: RSC Adv. 2025 Jul 15;15(30):24917–29. doi: 10.1039/d5ra03128a (PMC12261076; doi:10.1039/d5ra03128a)
Supplement: RA-015-D5RA03128A-s001 [file RA-015-D5RA03128A-s001.pdf]

Supplementary Information (SI) for RSC Advances.
This journal is © The Royal Society of Chemistry 2025

A portable smartphone-based electrochemical sensing platform for rapid and sensitive detection of creatinine in blood serum

Rifat Rayhan[1,3], Md. Inzamamul Haque Shishir[1,3], Md. Abdul Khaleque[1,2], Md. Ruhul Amin[1,2], Md. Romzan Ali[1,2], Mohamed Aly Saad Aly[4,5,1*], Sakib Mahmud Ayon[1], Rahman Saidur[6,7], Tan Han Kim[6], Md. Abu Zaed[6], Md. Zaved Hossain Khan[1,2*]

[1]Laboratory of Nano-bio and Advanced Materials Engineering (NAME), Jashore University of Science and Technology, Jashore 7408, Bangladesh

[2]Department of Chemical Engineering, Jashore University of Science and Technology, Jashore 7408, Bangladesh

[3]Department of Biomedical Engineering, Jashore University of Science and Technology, Jashore 7408, Bangladesh

[4]School of Electrical and Computer Engineering, Georgia Institute of Technology, Atlanta, GA 30332, USA

[5]Department of Electrical and Computer Engineering at Georgia Tech Shenzhen Institute (GTSI), Shenzhen, Guangdong, 518055, China

[6] Research Centre for Nanomaterials and Energy Technology (RCNMET), School of Engineering and Technology, Sunway University, Bandar Sunway, 47500 Selangor Darul Ehsan, Malaysia

[7] School of Engineering, Lancaster University, Lancaster, LA1 4YW, UK

[*]Corresponding Authors: Dr. Mohamed Aly Saad Aly, email: mohamed.alysaadaly@ece.gatech.edu; Dr. Md. Zaved Hossain Khan, email: zaved.khan@just.edu.bd.

1. Electrochemical detection of creatinine

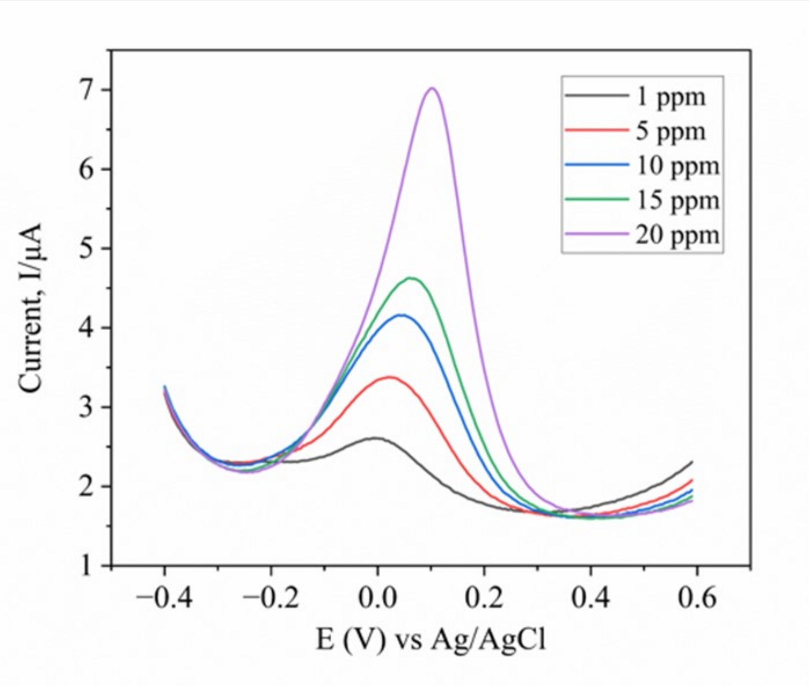

Figure S1. PPM optimization of standard copper solution.

2. Electrochemical polymerization

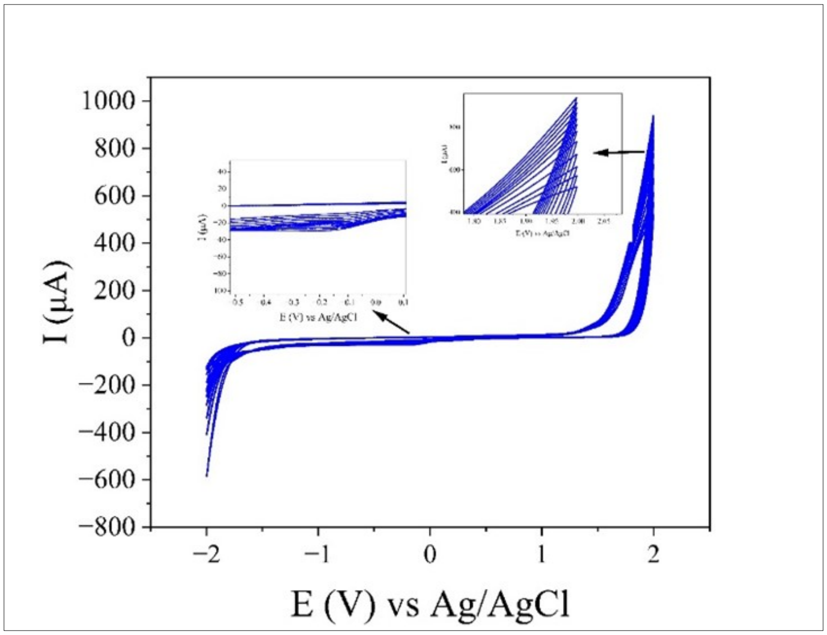

Figure S2. Cyclic voltammograms of Electrochemical polymerization of Ti3C2Tx@Poly-(L-Arg) nanocomposite on SPE electrode.

3. Stability, Reproducibility, and selectivity of the proposed sensor

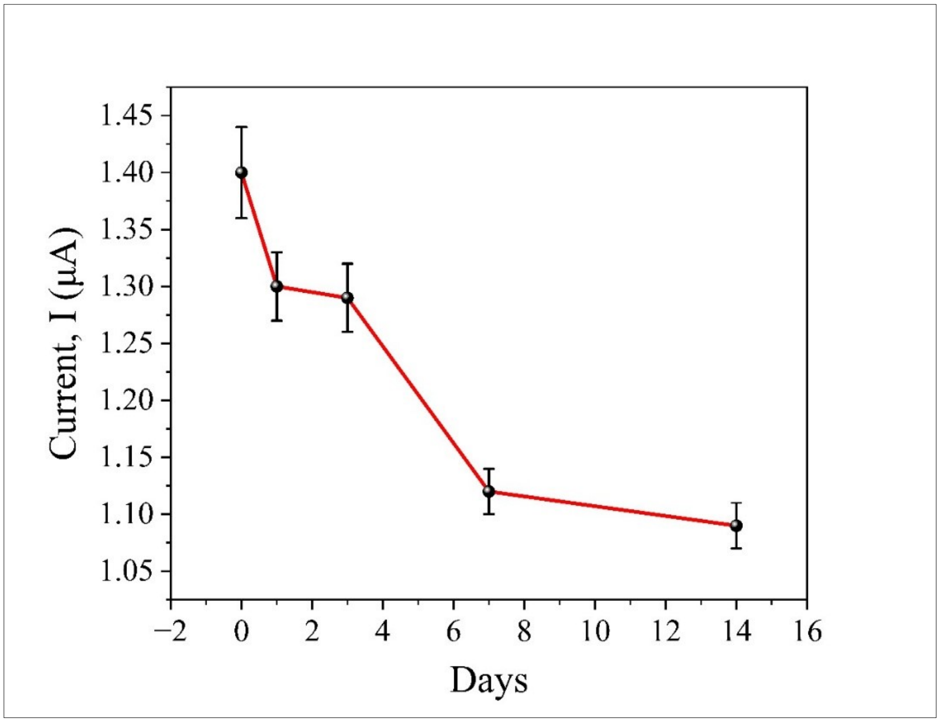

Figure S3. Stability of the proposed sensor.

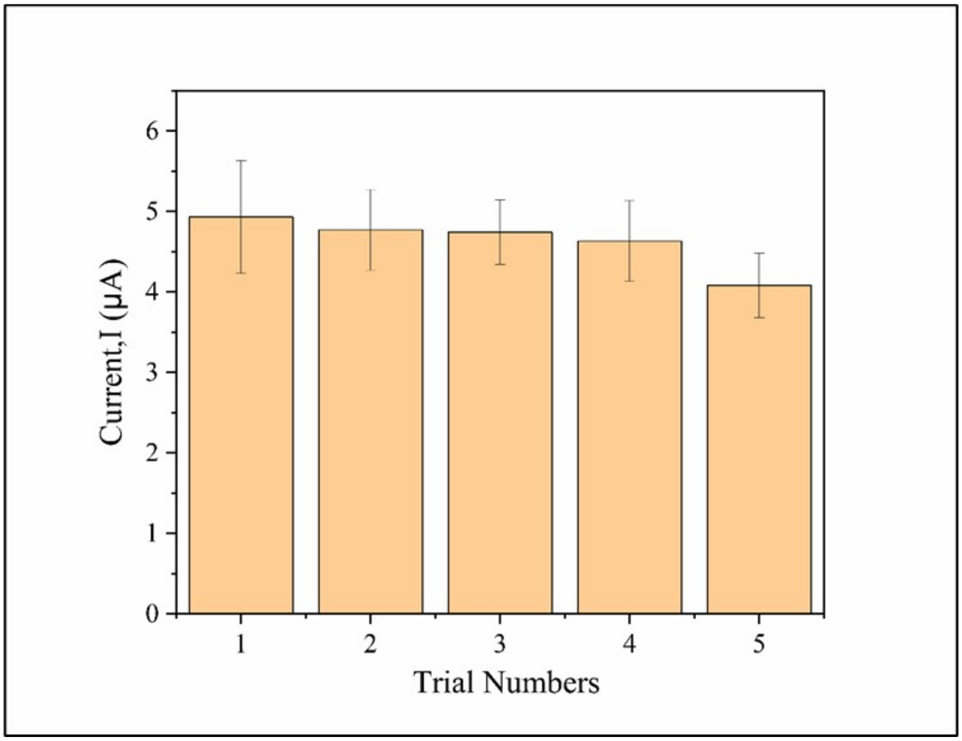

Figure S4. Reproducibility of the proposed sensor.

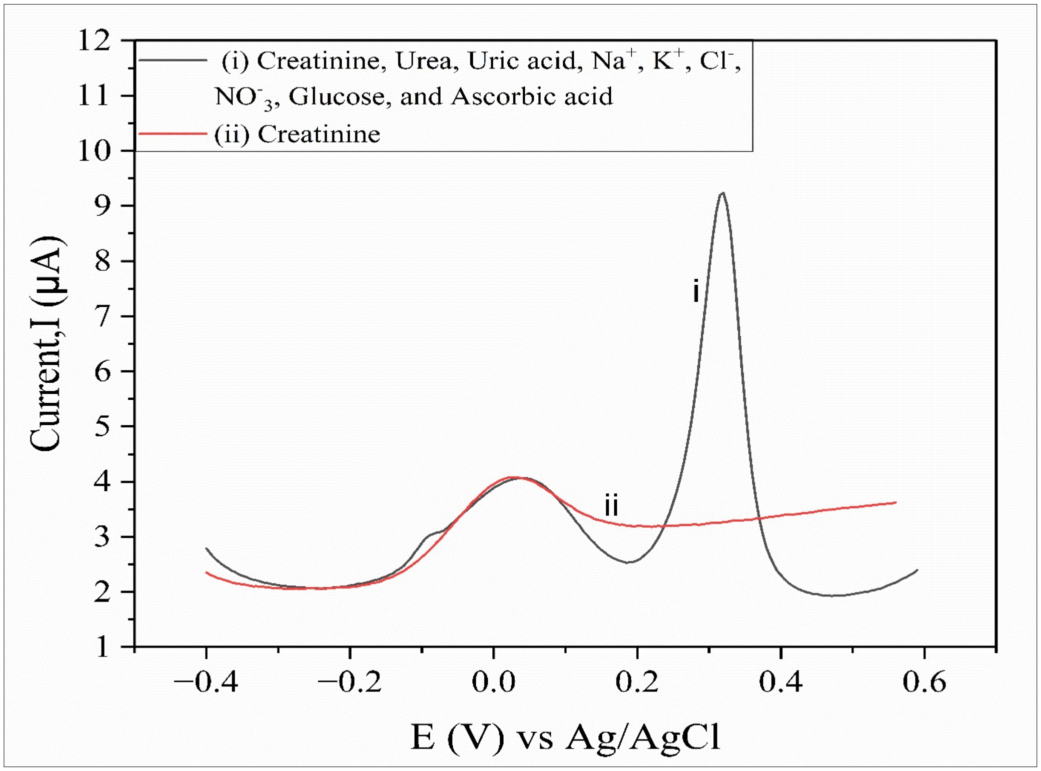

Figure S5. Selectivity of the proposed sensor

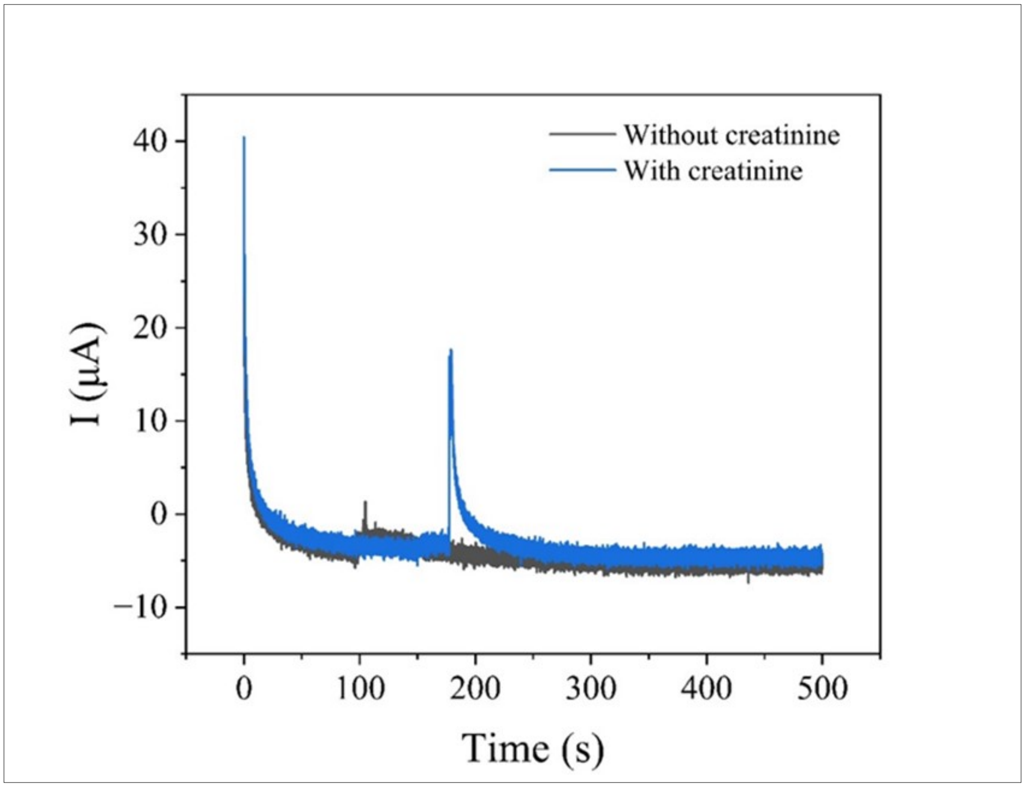

Figure S6. Chronoamperometric selectivity of creatinine sensor
